# The Experiences of Family Caregivers of Community-Dwelling Older Adults with Dementia in Providing Daily Oral Care

**DOI:** 10.1093/geroni/igab046.2945

**Published:** 2021-12-17

**Authors:** Abby Hellem, Kexin Zhou, Xi Chen, Jirakate Madiloggovit, Jennifer Nguyen, Rebecca Morris, Sato Ashida

**Affiliations:** University of Iowa, Iowa City, Iowa, United States

## Abstract

Individuals with dementia increasingly rely on caregivers for daily oral care over time. This study explored the experience of family caregivers of community-dwelling individuals with dementia in providing oral care and their interest in caregiver oral education using the concepts of Social Cognitive Theory. Twenty-three caregivers ages 19-80 participated in a semi-structured qualitative interview that also included a structured questionnaire. Majority of caregivers were female (83%) with an average age of 56 years; 29% were spouses. Fifty-four percent of care recipients had natural teeth only, 42% had teeth and dentures, and 4% had dentures only. Caregivers were generally knowledgeable about the importance of oral health, but some expressed a lack of knowledge in how to perform oral care for others. Caregivers reported high levels of outcome expectation, agreeing that providing oral care would improve care recipient’s oral health. Caregivers expressed mixed levels of self-efficacy; many cited reduced self-efficacy due to resistance or refusal of care. Quantitative data showed that higher confidence in knowledge and oral care skills was associated with greater confidence in providing oral care (r=0.726, p<0.001). Intent to participate in a caregiver oral health education program was associated with positive outcome expectations (r=0.73, p=0.007) and desire to learn the signs and symptoms of mouth pain and infection (r=0.72, p=0.009). Increasing family caregiver’s oral health knowledge and skills, outcome expectations, and self-efficacy to provide care may help improve the oral health of persons with dementia. Additional qualitative and quantitative data and implications for practice will be presented.

